# Association between time to colonoscopy after positive fecal testing and colorectal cancer outcomes in Alberta, Canada

**DOI:** 10.1177/09691413241239023

**Published:** 2024-03-15

**Authors:** Darren R Brenner, Chantelle Carbonell, Linan Xu, Nicole Nemecek, Huiming Yang

**Affiliations:** 1 Department of Oncology, 2129 University of Calgary, Calgary, AB, Canada; 2 Screening Programs, 3146 Alberta Health Services, Calgary, AB, Canada

**Keywords:** Colorectal cancer, cancer screening, fecal immunochemical test, colonoscopy, retrospective cohort study

## Abstract

**Objective:**

To quantify the associations between time to colonoscopy after a positive fecal immunochemical test (FIT+) and colorectal cancer (CRC)-related outcomes in the context of a provincial, population-based CRC screening program.

**Setting:**

Population-based, retrospective cohort study in Alberta, Canada, including Albertans aged 50–74 with at least one FIT+ in 2014–2017.

**Methods:**

Study outcomes were CRC diagnosis after a FIT+ and a diagnostic follow-up colonoscopy in 2014–2019 and CRC stage at diagnosis. Multivariable logistic regression models were used to evaluate the relative risk of any CRC or advanced-stage CRC. Results were presented as crude odds ratio (OR) and adjusted OR (aOR) with 95% confidence intervals (CIs).

**Results:**

Of the 787,967 participants who had a FIT, 63,232 (8%) had a FIT+ and met the study's eligibility criteria. The risk of any CRC or advanced-stage CRC stayed high and was relatively consistent for follow-up colonoscopies performed within 1–12 months of the FIT+. After 12 months, the risk of CRC was considerably higher, particularly for advanced-stage CRC. The OR and aOR for any CRC were 1.40 (95% CI: 1.13–1.73; *p* < 0.05) and 1.20 (95% CI: 0.96–1.49), respectively, and the OR and aOR for advanced-stage CRC were 1.42 (95% CI: 0.98–2.08) and 0.88 (95% CI: 0.59–1.32), respectively, for colonoscopy follow-up within 12–18 months versus 1–2 months.

**Conclusions:**

For Albertans who used FIT for CRC screening, a longer time interval between a FIT+ and follow-up colonoscopy, particularly over 12 months, increases the risk of having CRC and decreases the effectiveness of CRC screening programs.

## Introduction

Colorectal cancer (CRC) is the fourth most common cancer and the second leading cause of cancer-related deaths in Canada.^1^ Screening reduces CRC incidence and CRC-related morbidity and mortality through detecting and treating early stage cancers and removing precancerous polyps.^2–4^ The fecal immunochemical test (FIT) is widely used as an effective screening tool for CRC.^5^ A positive FIT (FIT+) result should be followed by a colonoscopy to identify and remove precancerous polyps or begin early treatment to reduce the risk of CRC-related mortality.^6^ According to Canadian CRC screening recommendations, a FIT should be performed every one to two years for individuals aged 50–74 who are at average risk (i.e. no personal or family history of CRC) and a FIT+ result should be followed by a colonoscopy within eight weeks.^7–9^ Delays in receiving a colonoscopy following a FIT+ result are influenced by several factors related to the patient, physician, and health system. The resulting longer time intervals between a FIT+ result and follow-up colonoscopy may potentially impact the effectiveness of provincial CRC screening programs. Furthermore, the coronavirus disease 2019 (COVID-19) pandemic and the subsequent health service interruptions have exacerbated these delays in many jurisdictions.^10^ To deal with growing colonoscopy wait times, many programs are moving to use FIT as the primary screening modality or as a triage tool for symptomatic patient referrals.^11,12^ In this study, we used Alberta data to quantify the associations between time from positive fecal testing to colonoscopy and CRC-related outcomes. A recent systematic review^13^ on this topic investigated three CRC-related outcomes: (a) CRC detection rate, (b) CRC stage at diagnosis, and (c) CRC-specific mortality. The Alberta Screening Programs database does not contain cancer-specific mortality information, so this study only evaluated the first two outcomes.

## Methods

### Study design and data sources

This retrospective cohort study in Alberta, Canada, used real-world, population-level data. Data sources for this study included the Alberta Provincial Cancer Screening database for fecal immunochemical tests and Alberta Health Care Insurance Plan (AHCIP) eligibility, the National Ambulatory Care Reporting System/Discharge Abstract Database for colonoscopy, the Alberta Cancer Registry database for identification of CRC, and the Alberta Postcode Translation File for Alberta Health Zones.

### Study population and eligibility criteria

The study population included Albertans aged 50–74 with at least one FIT+ in 2014–2017. Age was defined at the index FIT date. If the individual had multiple FIT+ in the target year, the most recent FIT was defined as the index FIT in the cohort. These analyses were focused on screening-related colonoscopies. To remove those who received diagnostic colonoscopies for symptomatic reasons, individuals were excluded if they were diagnosed with CRC or had a colonoscopy within 1–14 days of the index FIT+ result. Additionally, individuals were excluded if they had a prior history of CRC, became AHCIP ineligible within 12 months of index FIT+ result, had a colonoscopy within five years prior to the index FIT+ result date, had a FIT+ result within two years prior to the index FIT+ result date, or were diagnosed with CRC after the FIT+ result but prior to the first follow-up colonoscopy, which may be due to data entry/capture error or incomplete entries in the database.

### Follow-up time intervals and cancer outcomes

The study exposure was the time interval from the index FIT+ result to the follow-up colonoscopy. The reference group was 1–3 months, and comparison time intervals were 3–6 months, 6–9 months, 9–12 months, and more than 12 months. A single month was defined as 30.5 days. The reference group was selected to reflect the recommended interval that the colonoscopy be performed within eight weeks of a FIT+ test.^9^ Individuals who did not have a colonoscopy after the index FIT+ result by the end of 2019 were considered lost to follow-up. If the individual had multiple colonoscopies after the index FIT+ result, then the first colonoscopy was selected for this study to calculate the follow-up time interval (most recent colonoscopy and most recent FIT test). All follow-up colonoscopies were performed between 2014 and 2019. The follow-up ending time was lengthened due to COVID-19. The maximum time interval between the index FIT+ result and follow-up colonoscopy varied from 24 to 59 months to examine variability in long-term trends.

The primary outcome was CRC detection rate; specifically, CRC was diagnosed after the index FIT+ result and after the first follow-up colonoscopy between 2014 and 2019. The secondary outcome was the CRC stage at diagnosis, including both in situ and invasive CRC. Advanced-stage CRC was defined as stage III or stage IV carcinoma as per the American Joint Committee on Cancer (AJCC) Staging Manual.^14^

### Alberta Health Zones

We analyzed the risk of any CRC and advanced-stage CRC in five separate Alberta Health Zones. The Alberta Health Zones were defined as Zone 1: South Zone; Zone 2: Calgary Zone; Zone 3: Central Zone; Zone 4: Edmonton Zone; and Zone 5: North Zone.^15^

### Statistical analysis

Descriptive statistics (means and proportions) were estimated by month and relevant subgroups. Rates of CRC and advanced-stage CRCs were estimated per 1000 eligible individuals. Multivariable logistic regression models were used to evaluate the relative risk of any CRC or advanced-stage CRC. The results were presented as crude odds ratio (OR) and adjusted OR (aOR), and the corresponding 95% confidence intervals (CIs).

## Results

### Patient characteristics

**Figure 1.**
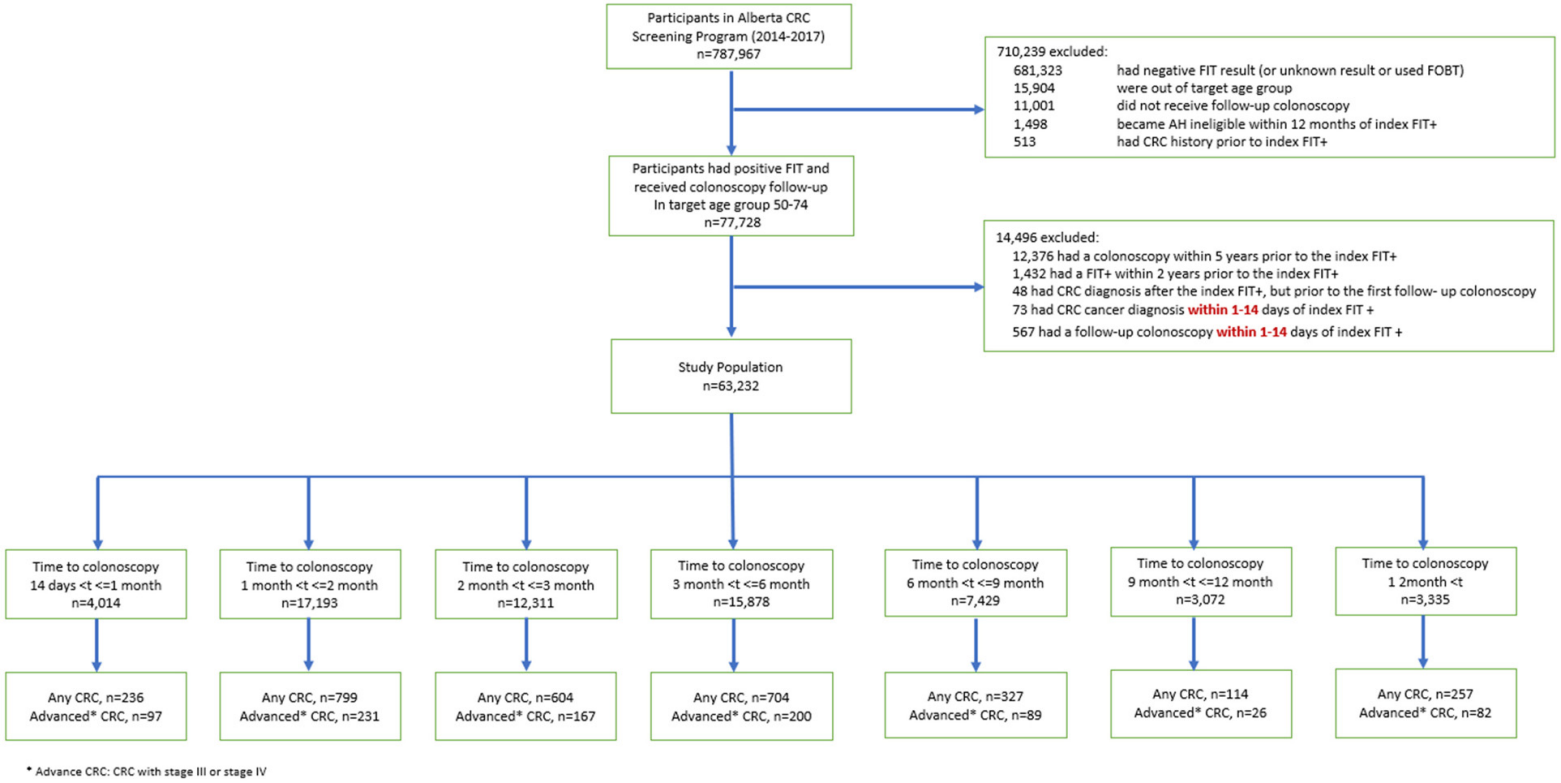
Study inclusion/exclusion of participants in Alberta's Colorectal Cancer Screening Program in 2014–2017 after applying relevant eligibility criteria.

Of the 787,967 participants in Alberta's CRC screening program, 77,728 (9.9%) were FIT+, received a follow-up colonoscopy, and were in the target age group of 50–74 (patient eligibility flow chart presented in Figure 1). Among this group, 12,376 individuals were excluded for having had a colonoscopy within five years prior to the index FIT+; 1432 for having had a FIT+ within two years prior to the index FIT+; 48 for CRC diagnosis after the index FIT+, but prior to the first follow-up colonoscopy; 73 for CRC diagnosis within 1–14 days of the index FIT+; and 567 for follow-up colonoscopy within 1–14 days of the index FIT+ (Figure 1). Of the remaining 63,232 FIT+ individuals aged 50–74 who received a follow-up colonoscopy, 4014 (6.3%) received a follow-up colonoscopy 14 days to 1 month after the index FIT+; 17,193 (27%) 1–2 months after the index FIT+; 12,311 (20%) 2–3 months after the index FIT+; 15,878 (25%) 3–6 months after the index FIT+; 7429 (12%) 6–9 months after the index FIT+; 3072 (4.9%) 9–12 months after the index FIT+; and 3335 (5.3%) received a follow-up colonoscopy more than 12 months after the index FIT+ (Figure 1 and Table 1). The mean (SD) age was 61 (6.9) years and 61% were male (Table 1).

**Table 1.** Baseline characteristics of participants stratified by the time to colonoscopy after positive fecal immunochemical test results in Alberta's Colorectal Cancer Screening Program in 2014–2017.

Characteristics	Total (*n* = 63,232)	14 days to 1 month (*n* = 4014)	1–2 months (*n* = 17,193)	2–3 months (*n* = 12,311)	3–6 months (*n* = 15,878)	6–9 months (*n* = 7429)	9–12 months (*n* = 3072)	>12 months (*n* = 3335)	*P* value
Mean age, years (SD)	61.3 (6.9)	61.3 (6.8)	61.1 (7.0)	61.4 (6.9)	61.4 (6.8)	61.2 (6.7)	61.3 (6.8)	61.3 (6.9)	0.0008
Age group, *n* (%)									0.0002
50–59	27,028 (42.7)	1682 (41.9)	7596 (44.2)	5203 (42.3)	6653 (41.9)	3189 (42.9)	1290 (42.0)	1415 (42.4)	
60–69	26,908 (42.6)	1767 (44.0)	7062 (41.1)	5210 (42.3)	6880 (43.3)	3200 (43.1)	1351 (44.0)	1438 (43.1)	
70–74	9296 (14.7)	565 (14.1)	2535 (14.7)	1898 (15.4)	2345 (14.8)	1040 (14.0)	431 (14.0)	482 (14.5)	
Male sex, *n* (%)	38,421 (60.8)	2441 (60.8)	10,529 (61.2)	7484 (60.8)	9559 (60.2)	4459 (60.0)	1857 (60.4)	2092 (62.7)	0.0889
Initial screen, *n* (%)	26,204 (41.4)	1774 (44.2)	7152 (41.6)	4923 (40.0)	6323 (39.8)	3058 (41.2)	1335 (43.5)	1639 (49.1)	<0.0001
Cancer year after 2017, *n* (%)	405 (0.6)	10 (0.2)	52 (0.3)	69 (0.6)	89 (0.6)	33 (0.4)	14 (0.5)	138 (4.1)	<0.0001
Alberta Health Zone, *n* (%)									<0.0001
Zone 1	5123 (8.1)	448 (11.2)	1818 (10.6)	1499 (12.2)	953 (6.0)	169 (2.3)	78 (2.5)	158 (4.7)	
Zone 2	20,039 (31.7)	1671 (41.6)	9353 (54.4)	4919 (40.0)	2858 (18.0)	502 (6.8)	213 (6.9)	523 (15.7)	
Zone 3	8156 (12.9)	874 (21.8)	2595 (15.1)	1916 (15.6)	1837 (11.6)	465 (6.3)	169 (5.5)	300 (9.0)	
Zone 4	23,177 (36.7)	472 (11.8)	1537 (8.9)	2519 (20.5)	8561 (53.9)	5723 (77.0)	2386 (77.7)	1979 (59.3)	
Zone 5	6737 (10.7)	549 (13.7)	1890 (11.0)	1458 (11.8)	1669 (10.5)	570 (7.7)	226 (7.4)	375 (11.2)	

### Time to colonoscopy and risk of CRC outcomes

**Figure 2.**
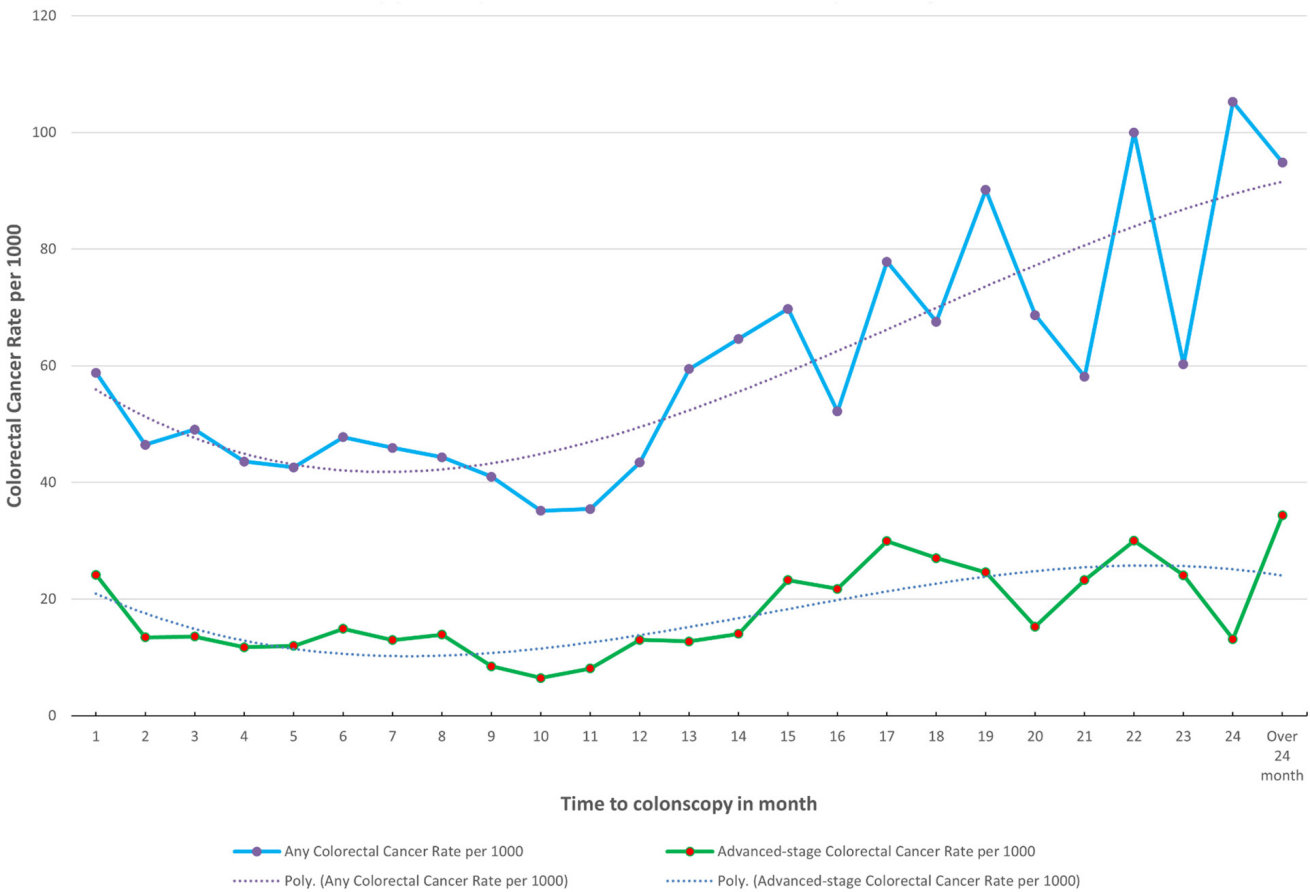
Time to colonoscopy after positive fecal immunochemical test results between 2014 and 2019 in Alberta by waiting time in months, up to 24 months.

The risk of having any CRC or advanced-stage CRC stayed high and was relatively consistent for follow-up colonoscopies performed within 1–12 months after the index FIT+ (Figure 2, Table 2, and Supplemental Figure 1). After 12 months, the risk of CRC was considerably higher, particularly for advanced-stage CRC (Figure 2 and Supplemental Table 1). Between the 12- to 13-month period and the 24-month period, the rate of any CRC and advanced-stage CRC increased (Figure 2 and Supplemental Table 1). In the 12- to 13-month period, the rate per 1000 for any CRC and advanced-stage CRC was 59 (95% CI: 38.09–80.80) and 13 (95% CI: 2.61–22.87), respectively (Supplemental Table 1). In the 24-month period, the rate per 1000 for any CRC and advanced-stage CRC was 95 (95% CI: 77.59–112.11) and 34 (95% CI: 23.60–45.05), respectively (Supplemental Table 1). Compared to follow-up colonoscopies performed within 1–2 months after the index FIT+, for 12–18 months, the OR and aOR for any CRC were 1.40 (95% CI: 1.13–1.73; *p* < 0.05) and 1.20 (95% CI: 0.96–1.49), respectively (Table 2). The OR and aOR for advanced-stage CRC for 12–18 months were 1.42 (95% CI: 0.98–2.08) and 0.88 (95% CI: 0.59–1.32), respectively (Table 2). The associations between time to colonoscopy and risk of any CRC and advanced-stage CRC differed somewhat across strata of age, gender, subsequent FIT versus initial FIT, year of cancer diagnosis (before 2017 vs. after 2017), and Alberta Health Zone (Table 3). Stronger associations were observed for any CRC and advanced-stage CRC among older individuals than younger individuals. Those among the 60–69 age group had aORs of 1.59 (95% CI: 1.46–1.73; *p* < 0.05) and 1.42 (95% CI: 1.22–1.65; *p* < 0.05) for any CRC and advanced-stage CRC, respectively, compared to the 50–59 age group (Table 3). Those among the 70–74 age group had aORs of 2.06 (95% CI: 1.85–2.29; *p* < 0.05) and 1.60 (95% CI: 1.31–1.95; *p* < 0.05) for any CRC and advanced-stage CRC, respectively, compared to the 50–59 age group (Table 3). A stronger association (*p* < 0.05) for any CRC was found among males compared to females (Table 3). Alberta Health Zones 3, 4, and 5 are associated with an increased risk of CRC compared to Zone 1 (*p* < 0.05) (Table 3).

**Table 2.** Risk of colorectal cancer stratified by the time to colonoscopy after FIT+ results in Alberta, up to over 12 months.

Time to colonoscopy	FIT+, n	Any colorectal cancer				Advanced-stage colorectal cancer			
		*n*	Rate per 1000 (95% CI)	OR (95% CI)	aOR^a^ (95% CI)	*n*	Rate per 1000 (95% CI)	OR (95% CI)	aOR^b^ (95% CI)
14 days to 1 month	4014	236	59 (51.52–66.07)	1.28^c^ (1.10–1.49)	1.23^c^ (1.06–1.44)	97	24 (19.41–28.92)	1.82^c^ (1.43–2.31)	1.79^c^ (1.40–2.28)
1–2 months	17,193	799	46 (43.33–49.62)	1 (–)	1 (–)	231	13 (11.71–15.16)	1 (–)	1 (–)
2–3 months	12,311	604	49 (45.25–52.88)	1.06 (0.95–1.18)	1.03 (0.93–1.15)	167	14 (11.52–15.61)	1.01 (0.83–1.23)	0.93 (0.76–1.14)
3–6 months	15,878	704	44 (41.14–47.54)	0.95 (0.86–1.06)	0.87^c^ (0.77–0.97)	200	13 (10.86–14.33)	0.94 (0.77–1.13)	0.76^c^ (0.62–0.94)
6–9 months	7429	327	44 (39.35–48.68)	0.95 (0.83–1.08)	0.82^c^ (0.71–0.95)	89	12 (9.51–14.45)	0.89 (0.70–1.14)	0.69^c^ (0.53–0.91)
9–12 months	3072	114	37 (30.42–43.79)	0.79^c^ (0.65–0.97)	0.68^c^ (0.55–0.84)	26	8 (5.22–11.70)	0.63^c^ (0.42–0.94)	0.47^c^ (0.31–0.72)
12–18 months	3335	257	77 (68.01–86.11)	1.40^c^ (1.13–1.73)	1.20 (0.96–1.49)	82	25 (19.33–29.84)	1.42 (0.98–2.08)	0.88 (0.59–1.32)
18–24 months				1.79^c^ (1.32–2.43)	1.59^c^ (1.17–2.17)			1.63 (0.93–2.87)	0.83 (0.45–1.52)
Over 24 months				2.15^c^ (1.74–2.66)	1.80^c^ (1.45–2.24)			2.61^c^ (1.84–3.70)	0.87 (0.58–1.30)
Overall	63,232	3041	48 (46.43–49.76)			892	14 (13.19–15.03)		
Continuous time, in months				1.02^c^ (1.01–1.02)	1.02^c^ (1.01–1.02)			1.02 (1.01–1.03)	0.99 (0.98–1.00)

aOR: adjusted odds ratio; CI: confidence interval; FIT+, positive fecal immunochemical test; OR: odds ratio.

^a^ Adjusted for age, initial screen, gender, and patient's geographic region (Alberta Health Zone).

^b^ Adjusted for age, initial screen, gender, and patient's geographic region (Alberta Health Zone), and cancer diagnosis year.

^c^ *P* value <0.05.

**Table 3.** Association between baseline characteristics and risk of any colorectal cancer or advanced-stage colorectal cancer in the multiple logistic regression.

Characteristics	Any colorectal cancer		Advanced-stage colorectal cancer	
	aOR^a^	95% CI	aOR^b^	95% CI
Age group				
50–59	1	—	1	—
60–69	1.59^c^	1.46–1.73	1.42^c^	1.2–1.7
70–74	2.06^c^	1.85–2.29	1.60^c^	1.3–2.0
Gender: Female versus male	0.917^c^	0.85–0.99	0.95	0.83–1.1
FIT + subsequent screen versus initial screen	0.55^c^	0.51–0.59	0.50^c^	0.44–0.58
Cancer year before 2017 versus after 2017	—	—	0.04^c^	0.028–0.046
Alberta Health Zone				
Zone 1	1	—	1	—
Zone 2	1.16	1.00–1.36	1.13	0.85–1.50
Zone 3	1.26^c^	1.06–1.50	1.15	0.83–1.58
Zone 4	1.21^c^	1.04–1.41	1.31	0.99–1.74
Zone 5	1.12^c^	0.93–1.34	1.28	0.92–1.77

aOR: adjusted odds ratio; CI: confidence interval; OR: odds ratio.

^a^ Adjusted for time to colonoscopy, age, initial screen, gender, and patient's geographic region (Alberta Health Zone).

^b^ Adjusted for time to colonoscopy, age, initial screen, gender, and patient's geographic region (Alberta Health Zone), and cancer diagnosis year.

^c^ *P* value <0.05.

## Discussion

This study aimed to quantify the associations between time from positive fecal testing to colonoscopy and CRC-related outcomes in a real-world population using data from Alberta, Canada. In this large population-based setting, the risk of having any CRC or advanced-stage CRC remained high post-FIT+ test and was relatively consistent for follow-up colonoscopies performed within 1–12 months after the index FIT+. After 12 months, the risk of CRC increased considerably, particularly for advanced-stage CRC. We observed that for the 12- to 13-month period, the rate per 1000 for any CRC and advanced-stage CRC was 59 (95% CI: 38.09–80.80) and 13 (95% CI: 2.61–2.87), respectively. Previous population-based studies by Corley et al.^16^ and Lee et al.^17^ observed similar findings of higher CRC incidence associated with colonoscopy after 12 months compared with the baseline time period, ranging from 76 to 98 per 1000 persons. They also reported significantly higher rates of advanced-stage CRC after 12 months, with a rate of 31 per 1000.^16,17^ Furthermore, the aORs associated with colonoscopy after 12 months in these studies ranged from 2.17 (95% CI: 1.44–3.26) to 2.25 (95% CI: 1.86–2.68) for any CRC and 2.84 (95% CI: 1.43–5.64) to 3.22 (95% CI: 2.44–4.25) for advanced-stage CRC, which were greater than our findings of the 1.20 (95% CI: 0.96–1.49) aOR for any CRC and the 0.88 (95% CI: 0.59–1.32) aOR for advanced-stage CRC for 12–18 months compared with the baseline time period.^16,17^ Our study population included a total of 63,232 individuals, similar in size to Corley et al.'s^16^ 70,124 included individuals and larger than Lee et al.'s^17^ 39,346 included individuals. Therefore, our results support previous observations that a prolonged time interval between the index FIT+ and a follow-up colonoscopy, particularly over 12 months, may result in CRC stage shift, and therefore decrease the population-level effectiveness of CRC screening programs.^13^ Interestingly, Corley et al. also observed a significant increase in incidence for any CRC and advanced-stage CRC associated with colonoscopy after nine months, compared to within one month, after a FIT+ result, with aORs of 1.48 (95% CI: 1.05–2.08) and 1.55 (95% CI: 1.05–2.28), respectively.^16^ A 2020 study of over 100,000 individuals also reported a significant increase in CRC incidence associated with colonoscopy after nine months, with aORs of 1.75 (95% CI: 1.15–2.67) for any CRC and 2.79 (95% CI: 1.03–7.57) for advanced-stage CRC.^18^ There was one study that reported a significantly higher incidence of any CRC and advanced-stage CRC after 3 months compared to within 1 month, with aORs of 5.32 (95% CI: 4.89–5.79) and of 2.59 (95% CI: 2.19–3.06).^19^ In contrast, another study observed no significant associations between either incidence of any CRC or advanced-stage CRC and a colonoscopy after 6 months; however, this cohort was much smaller with only 2362 individuals.^20^ Given these mixed findings, future research is still needed to gain a better understanding of the associations between time from positive fecal testing to colonoscopy and CRC-related outcomes in real-world populations.

Compared to Alberta Health Zone 1, Alberta Health Zones 3, 4, and 5, on average, had longer wait times which resulted in an increased risk of CRC by zone. This study population included any Albertans aged 50–74 with at least one FIT+ in 2014–2017. The FIT only became available in Alberta in November of 2013.^21^ Thus, higher CRC diagnoses captured in this time frame may reflect cancers being diagnosed early due to the province's newly available FIT screening modality.

In Alberta, the expected wait time for a screening colonoscopy for individuals at average risk for CRC is >24 months.^11^ This time interval from a FIT+ to colonoscopy in Alberta is 12-fold longer than the Canadian CRC screening recommendation of receiving a follow-up colonoscopy within 8 weeks following a FIT+ result.^7–9^ Therefore, provincial screening programs support using the FIT as the primary screening method instead of colonoscopy for those at average risk for CRC.^11^ An increase in the use of FIT and reduced use of colonoscopy will maximize available resources and decrease extensive waiting periods.^22^ Furthermore, a FIT is non-invasive and can be easily performed in one's private home.^8^ By encouraging the use of FIT as the primary screening method, colonoscopies will be reserved for individuals at higher risk of having CRC, including people with a family history of CRC, or for diagnostic follow-up of people with a FIT+ result or surveillance post polypectomy.^8^

The strengths of this study include its large size and the leveraging of population-based data, which captures information on all eligible individuals in the province of Alberta. The use of population-based data may have minimized the risk of selection biases and increased the degree of generalizability of these results. As this study was observational in nature, biases may exist related to non-interventional studies such as patient referral practices that we are not able to account for. Further limitations include changes to the staging data collection system mandated by Statistics Canada that were implemented nationwide for 2018 case incidence and forward. Effective for 2018 cases onward, the collection system used is Tumor Node Metastasis AJCC 8th edition versus the historic Collaborative Stage for incidence years 2004–2017 inclusive. Thus, caution should be used when comparing data collected from 2004 to 2017 to data collected from 2018 onwards. To minimize the impact of these cancer staging data system changes, we added an adjusted variable cancer diagnosis year (diagnosed before 2017 vs. after 2017) into the modeling.

## Conclusions

Among the screening participants in Alberta, the risk of any CRC or advanced-stage CRC remained consistently high for follow-up colonoscopies performed within 1–12 months after the index FIT+. After 12 months, the risk of CRC was significantly elevated, particularly for advanced-stage cases. These findings suggest that, in screening-eligible patients, a longer time interval between a FIT+ and follow-up colonoscopy, particularly over 12 months, decreases the population-level effectiveness of CRC screening programs.

## Supplemental Material

sj-docx-1-msc-10.1177_09691413241239023 - Supplemental material for Association between time to colonoscopy after positive fecal testing and colorectal cancer outcomes in Alberta, Canada Supplemental material, sj-docx-1-msc-10.1177_09691413241239023 for Association between time to colonoscopy after positive fecal testing and colorectal cancer outcomes in Alberta, Canada by Darren R Brenner, Chantelle Carbonell, Linan Xu, Nicole Nemecek and Huiming Yang in Journal of Medical Screening
