# Supplementary material for: Association between time to colonoscopy after positive fecal testing and colorectal cancer outcomes in Alberta, Canada
Source: J Med Screen. 2024 Mar 15;31(4):232–8. doi: 10.1177/09691413241239023 (PMC11526417; doi:10.1177/09691413241239023)

**Association between time to colonoscopy after positive fecal testing and colorectal cancer outcomes in Alberta, Canada**

Darren R. Brenner^1*^, Chantelle Carbonell^1^, Linan Xu^2^, Nicole Nemecek^2^, Huiming Yang^2^

Affiliations:

1. Department of Oncology, University of Calgary, Calgary, AB, Canada
2. Screening Programs, Alberta Health Services, Calgary, AB, Canada

*Corresponding Author:
Darren R. Brenner
Department of Oncology and Community Health Sciences
Cumming School of Medicine, University of Calgary,
Health Research Innovation Centre Room 2AA21
3230 Hospital Dr NW, Calgary, AB,
T2N 4Z6, Canada

[darren.brenner@ucalgary.ca](mailto:darren.brenner@ucalgary.ca)

The authors declare no potential conflicts of interest.

**SUPPLEMENTAL FILE**

Supplemental Table 1. Risk of colorectal cancer stratified by the time to colonoscopy after positive fecal immunochemical test results in Alberta, up to over 24 months.

Supplemental Figure 1. Cubic spline curve from the adjusted logistic model.

**Supplemental Table 1. Risk of colorectal cancer stratified by the time to colonoscopy after positive fecal immunochemical test results in Alberta, up to over 24 months.**

| Time to colonoscopy | Positive FIT, n | Any colorectal cancer | | | Advanced-stage colorectal cancer | | |
| --- | --- | --- | --- | --- | --- | --- | --- |
|  |  | n | Rate per 1000 | | n | Rate per 1000 | |
|  |  |  | Rate | 95% CI |  | Rate | 95% CI |
| 14 days - 1 month | 4014 | 236 | 59 | 51.52–66.07 | 97 | 24 | 19.41–28.92 |
| 1 month - 2 months | 17,193 | 799 | 46 | 43.33–49.62 | 231 | 13 | 11.71–15.16 |
| 2 months - 3 months | 12,311 | 604 | 49 | 45.25–52.88 | 167 | 14 | 11.52–15.61 |
| 3 months - 4 months | 7157 | 312 | 44 | 38.86–48.32 | 84 | 12 | 9.24–14.23 |
| 4 months - 5 months | 4765 | 203 | 43 | 36.87–48.34 | 57 | 12 | 8.88–15.05 |
| 5 months - 6 months | 3956 | 189 | 48 | 41.13–54.42 | 59 | 15 | 11.14–18.69 |
| 6 months - 7 months | 3005 | 138 | 46 | 38.44–53.41 | 39 | 13 | 8.93–17.03 |
| 7 months - 8 months | 2302 | 102 | 44 | 35.90–52.72 | 32 | 14 | 9.12–18.68 |
| 8 months - 9 months | 2122 | 87 | 41 | 32.56–49.44 | 18 | 8 | 4.58–12.38 |
| 9 months - 10 months | 1394 | 49 | 35 | 25.48–44.82 | 9 | 6 | 2.25–10.66 |
| 10 months - 11 months | 987 | 35 | 35 | 23.92–47.00 | 8 | 8 | 2.51–13.70 |
| 11 months - 12 months | 691 | 30 | 43 | 28.22–58.61 | 9 | 13 | 4.57–21.48 |
| 12 months - 13 months | 471 | 28 | 59 | 38.09–80.80 | 6 | 13 | 2.61–22.87 |
| 13 months - 14 months | 356 | 23 | 65 | 39.07–90.14 | 5 | 14 | 1.82–26.27 |
| 14 months - 15 months | 258 | 18 | 70 | 38.68–100.85 | 6 | 23 | 4.87–41.65 |
| 15 months - 16 months | 230 | 12 | 52 | 23.43–80.91 | 5 | 22 | 2.89–40.59 |
| 16 months - 17 months | 167 | 13 | 78 | 37.21–118.48 | 5 | 30 | 4.09–55.79 |
| 17 months - 18 months | 148 | 10 | 68 | 27.13–108.01 | 4 | 27 | 0.90–53.15 |
| 18 months - 19 months | 122 | 11 | 90 | 39.34–140.99 | 3 | 25 | -2.89–52.07 |
| 19 months - 20 months | 131 | 9 | 69 | 25.39–112.02 | 2 | 15 | -5.73–36.26 |
| 20 months - 21 months | 86 | 5 | 58 | 8.68–107.60 | 2 | 23 | -8.60–55.11 |
| 21 months - 22 months | 100 | 10 | 100 | 41.20–158.80 | 3 | 30 | -3.43–63.43 |
| 22 months - 23 months | 83 | 5 | 60 | 9.05–111.43 | 2 | 24 | -8.89–57.09 |
| 23 months - 24 months | 76 | 8 | 105 | 36.27–174.26 | 1 | 13 | -12.46–38.78 |
| Over 24 months | 1107 | 105 | 95 | 77.59–112.11 | 38 | 34 | 23.60–45.05 |
| Overall | 63232 | 3041 | 48 | 46.43–49.76 | 892 | 14 | 13.19–15.03 |

CI, confidence interval; FIT, fecal immunochemical test.

**Supplemental Figure 1. Cubic spline curve from the adjusted logistic model.**


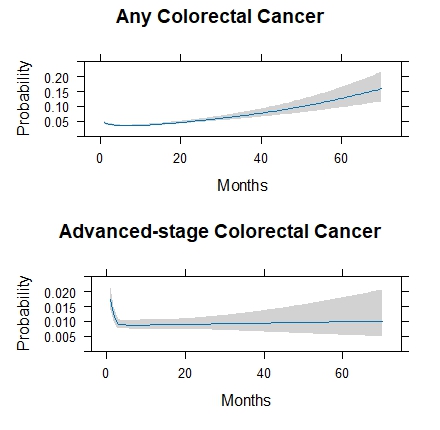

Supplement: sj-docx-1-msc-10.1177_09691413241239023 - Supplemental material for Association between time to colonoscopy after positive fecal testing and colorectal cancer outcomes in Alberta, Canada [file sj-docx-1-msc-10.1177_09691413241239023.docx]
